# Meta-analysis of subxiphoid approach versus lateral approach for thoracoscopic Thymectomy

**DOI:** 10.1186/s13019-020-01135-w

**Published:** 2020-05-12

**Authors:** Jiaduo Li, Guoyan Qi, Yaling Liu, Xuguang Zheng, Xiaohe Zhang

**Affiliations:** 1 Center of Treatment of Myasthenia Gravis Hebei Province, First Hospital of Shijiazhuang, Fangbei road No. 9, Shijiazhuang, 050011 Hebei Province China; 2 Department of Gastroenterology, First Hospital of Shijiazhuang, Shijiazhuang, China

**Keywords:** VATS, Subxiphoid, Lateral approach, Thoracoscopic thymectomy

## Abstract

**Background:**

Compared with traditional open surgery for thymectomy, video-assisted thoracoscopic surgery (VATS) reduces hospital stay, decreases postoperative pain, and recovers faster. VATS has become increasingly popular in the past decade. VATS techniques to perform a thymectomy include subxiphoid video-assisted thoracoscopic surgery (SVATS) or lateral video-assisted thoracoscopic surgery (LVATS). In this study, our objective was to systematically review on VATS thymectomy and draw a meta-analysis on the outcomes between the two approaches.

**Methods:**

We searched online databases and identified studies from database inception to 2019 that compared SVATS to LVATS thymectomy. Study endpoints included operative time, operative blood loss, length of hospital stay, postoperative pleural drainage, postoperative complications, conversion to open, oncologic outcomes.

**Results:**

Four hundred seventy-one patients were included in this study, for which 200 and 271 patients underwent SVATS and LVATS thymectomy, respectively. Patients in the SVATS group had significantly less operative time, operative blood loss, length of hospital stay, and postoperative complications were identified. There was no statistical difference in postoperative pleural drainage, conversion to open and oncologic outcomes. No hospital deaths were recorded for either procedure.

**Conclusions:**

While randomized controlled studies are required to make definitive conclusions, this meta-analysis suggests that SVATS thymectomy is safe and can achieve good and safe operative and perioperative outcomes similar or better to LVATS thymectomy.

## Introduction

Thymectomy is one of the most important treatments for patients with myasthenia gravis (MG) or thymoma. In recent years, the development of surgical techniques has led to the rapid development of surgical approaches to thymectomy. Now, VATS thymectomy through the intercostal approach has been the commonly used minimally invasive surgical procedure for thymus surgery and is applied worldwide [1–4]. SVATS is a newer alternative to LVATS and current research suggests that subxiphoid thoracoscopic thymectomy leads to less invasive than the lateral approach [5–9]. However, many surgeons remain reluctant to adopt SVATS surgical techniques for the treatment of patients with myasthenia gravis and thymomas for several reasons. The choice of SVATS or LVATS thymectomy remains controversial. There were no systematic reviews and meta-analysis to compare SVATS thymectomy with LVATS thymectomy. The purpose of this study was to determine whether SVATS thymectomy is a better approach to the operative and perioperative outcomes. We systematically identified and evaluated the existing data comparing the clinical outcomes of SVTAS thymectomy to LVATS thymectomy by using the techniques of meta-analysis.

## Methods

### Search strategy

Electronic database searches were performed with PubMed, Ovid Medline, Scopus, and Google Scholar of Abstract of Review of Effectiveness from database inception to Nov 2019. We used medical subject headings (MeSH) and free-text words. Search terms used included the following: thymectomy (MeSH), myasthenia gravis (MeSH), thymoma (MeSH), thymus gland (MeSH), thoracic surgery, video-assisted (MeSH), thoracoscopy (MeSH), subxiphoid, lateral, right and left. Only English language papers were included.

To achieve maximum sensitivity, all search terms were combined with Boolean operators and searched as both keywords and MeSH terms. After exclusion of articles based on title or abstract, full-text articles selected had reference lists searched for any potential further articles to be included in this review.

### Study selection

Inclusion criteria were met if: (I) the English language journal article described VATS thymectomy, (II) Randomized clinical trials (RCTs) or non-randomized controlled studies or observational studies (with at least 10 patients undergoing each intervention), comparing the subxiphoid approach with lateral approach for thoracoscopic thymectomy.

**Fig. 1:**
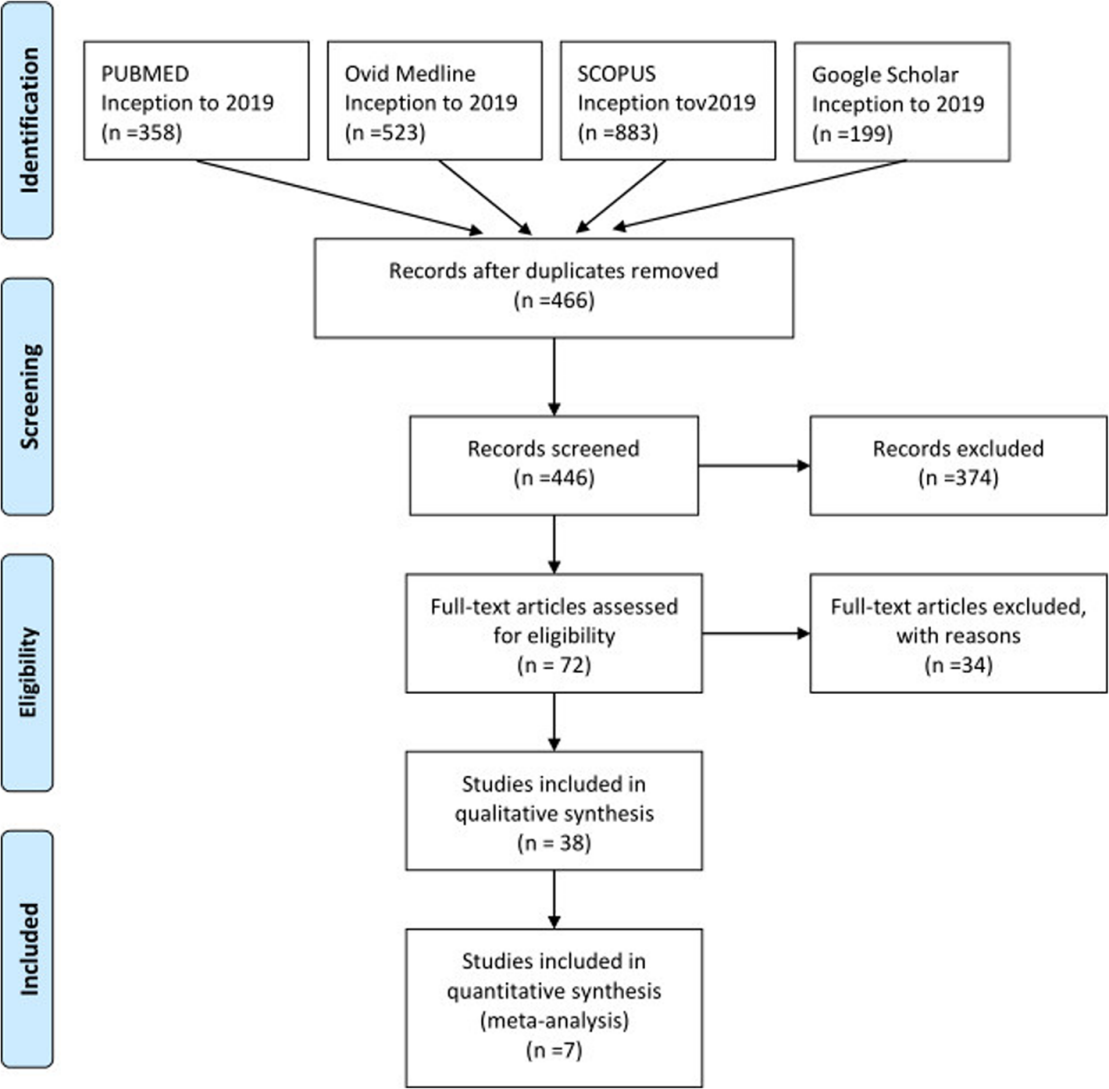
Flow diagram for study selection

Exclusion criteria were met if: (I) publication was not in English, (II) publication was not a journal article (book, book chapter), (III) publication was not about VATS thymectomy, (IV) publication was a health technology assessment that was not published in a peer-reviewed journal, (V) study was a review, (VI) publication did not have a comparator, (VII) publication was about da Vinci thymectomy, (VIII) the study did not provide quantitative results for at least one of the findings relative to the outcomes of interest. The PRISMA flowchart is outlined in Fig. 1.

### .Quality evaluation

We applied the Newcastle-Ottawa scale (NOS) [5] to evaluate the qualities of the included studies. A “star system” was used to judge the data quality of these studies based on three broad categories: the selection, the comparability, and the outcome or exposure of interest. The stars were summed to compare the quality of a study in a quantitative fashion. The scores ranged from 0 to 9 stars. Studies with scores of 6 stars or greater were considered to be of high-quality studies. Two reviewers (Yaling Liu and Jiaduo li) independently evaluated and cross-checked the qualities of the included studies, and assessed the bias of the studies. An open discussion was held to confirm the scores of those studies that received a different score from each reviewer.

### Data extraction

One investigator independently reviewed each included article under the guidance of two faculty members from the same center. Study endpoints included some or all of the following: age (years), gender, approach (subxiphoid or lateral), mean operative time (minutes), mean operative blood loss (milliliters [mL]), converted to open, postoperative pleural drainage (days), postoperative complication, length of hospital stay (days), oncologic outcomes.

### Statistical analysis

Conventional descriptive statistics were used to summarize the baseline demographics of the included patients. Data were presented as raw numbers, percentages, or means with standard deviations unless otherwise indicated. For dichotomous data (eg, incidence of complications), we used both a fixed and random effects model to calculate a pooled RR. For continuous data (eg, mean operating time), we used both a fixed and random effects model to calculate a weighted MD (WMD). In the case of continuous data presented as median and range, we estimated the mean and standard deviation according to the method described by Higgins [6, 7]. Heterogeneity was investigated by the use of the X2 test and I2 statistics. For I2 of between 0 and 25%, heterogeneity was considered as probably not important, between 25 and 50% moderate, between 50 and 90% (or if the *P*-value of X2 was < 0.10) substantial, and between 75 and 100% considerable [8]. If heterogeneity existed (> 25%), we analyzed data using a random-effects model. If heterogeneity was not important, a fixed-effects model was used. *P* < 0.05 was considered statistically significant.

All statistical analyses were performed by using Stata Statistical Software (Version 11.0; StataCorp LP, Texas, USA).

## Results

We identified 7 references through the aforementioned search criteria [8–15]. A total of 7 studies, with publication dates ranging from 2004 to 2019, contained pertinent perioperative outcome information regarding VATS thymectomy for myasthenia gravis and thymomas. The screening process for the studies is shown in Fig. 1, and the characteristics of the included studies are summarized in Table 1. All the included articles were nonrandomized and retrospective. The total number of patients was 670; of these patients, 312 were treated with SVATS thymectomy, and 358 received LVATS thymectomy.

**Table 1:** Summary of Study Demographics

Author	No.	No.	No. Male	No. Male	Mean ± SD	Mean ± SD	No. Myasthenia	No. Myasthenia	No. Thymoma	No. Thymoma
	SVATS	LVATS	SVATS	LVATS	Age SVATS	Age LVATS	Gravis SVATS	Gravis LVATS	SVATS	LVATS
Suda Takashi	46	35	23	15	53.9 ± 14.4	49.7 ± 17.8	11	10	11	27
Hsu C-P	15	12	5	7	41.2(23–80)	39.3(27–62)	15	12	4	2
Wang, H	36	47	NA	NA	NA	NA	0	0	36	47
Yano Motoki	14	46	7	20	48(18–79)	58(13–87)	1	2	7	26
Lu Qiang	41	36	16	13	36.3 ± 8.2	38.5 ± 9.1	1	2	10	12
Zhang ouqian	28	70	16	26	58.2 ± 10	54.8 ± 8.6	0	0	28	70
Yong Tang	20	25	NA	NA	NA	NA	NA	NA	NA	NA

NA, not available

Demographics were calculated using the subset of 7 comparative studies, and the characteristics of the included studies are summarized in Tables 2, 3, 4 and 5. Quality assessment of all studies was performed by using the NOS method (Table 6).

**Table 2:** Operative Outcomes for Thymectomy

Author	Mean ± SD Operation Time	Mean ± SD Operation Time	Mean ± SD Blood Loss	Mean ± SD Blood Loss	No. Converted to Open	No. Converted to Open
	SVATS, min	LVATS, min	SVATS, ml	LVATS, ml	SVATS	LVATS
Suda Takashi	140.3 ± 50.5	160.0 ± 57.2	2.2 ± 0.8	23.1 ± 22	0	0
Hsu C-P	151.3 ± 23.2	171.5 ± 32.1	NA	NA	0	0
Wang, H	54 ± 16	65 ± 18	51 ± 34	53 ± 44	2	2
Yano Motoki	154 ± 104	200 ± 119	89 ± 316	42 ± 80	0	0
Lu Qiang	95.3 ± 25.5	120 ± 24.6	25.5 ± 10.6	55.1 ± 10.4	2	4
Zhang Louqian	104 ± 29	116 ± 36	46.6 ± 30.9	50 ± 38.6	0	0
Yong Tang	136.1 ± 51.7	139.5 ± 39.7	66.5 ± 42.8	138.8 ± 123	NA	NA

**Table 3:** In-Hospital Postoperative Outcomes

Author	No. Postoperative complications	No. Postoperative complications	Mean ± SD Postoperative Pleural Drainage	Mean ± SD Postoperative Pleural Drainage	Mean ± SD Hospital Stay	Mean ± SD Hospital Stay
	SVATS	LVATS	SVATS, d	LVATS, d	SVATS	LVATS
Suda Takashi	2/46	2/35	NA	NA	4 ± 1.5	5.3 ± 2.3
Hsu C-P	0/15	0/12	3.1 ± 0.5	3.8 ± 0.6	NA	NA
Wang, H	5.40%	6.70%	1.3 ± 0.6	2.5 ± 1.0	2.2 ± 0.9	3.7 ± 1.3
Yano Motoki	1/14	7/39	1.0 ± 0	1.4 ± 0.8	5.4 ± 5.1	6.6 ± 5.7
Lu Qiang	1/41	1/36	1.8 ± 1.6	2.1 ± 1.3	3.6 ± 1.3	7.4 ± 2.3
Zhang Louqian	0/28	5/65	0 ± 1.0	4.6 ± 1.3	3.6 ± 1.2	4.3 ± 1.6
Yong Tang	NA	NA	1.0 ± 0.4	1.7 ± 1.0	3.8 ± 1.1	10.2 ± 17.5

NA, not available

**Table 4:** Postoperative Outcomes

Author	Postoperative Pain	Postoperative Pain	Radicality of the resection	Radicality of the resection	Remission of MG	Remission of MG
	SVATS	LVATS	SVATS, d	LVATS, d	SVATS	LVATS
Suda Takashi	Low	High	R0	R0	NA	NA
Hsu C-P	NA	NA	R0	R0	6/15	4/15
Wang, H	Low	High	NA	NA	NA	NA
Yano Motoki	NA	NA	R0	R0	NA	NA
Lu Qiang	Low	High	R0	R0	NA	NA
Zhang Louqian	Low	High	NA	NA	–	–
Yong Tang	NA	NA	–	–	NA	NA

NA, not available

**Table 5:** Summary of Oncologic outcomes

	SVATS					LVATS				
Study	Thymoma	Tumor diameter (cm)	Masaoka staging	MG	Carcinoma	Thymoma	Tumor diameter (cm)	Masaoka staging	MG	Carcinoma
Suda Takashi	11/46	NA	Stage I and II	11/46	0/46	27/35	NA	Stage I and II	10/35	0/35
Hsu C-P	4/11	≤4 cm	Stage I and II	15/15	0/15	2/10	≤4 cm	Stage I and II	12/12	0/12
Wang, H	36/36	NA	Stage I and II	0/36	NA	47/47	NA	Stage I and II	0/47	NA
Yano Motoki	7/14	4.5 ± 2.7	NA	1/14	2/14	26/46	NA	NA	2/46	2/46
Lu Qiang	10/31	≤5 cm	Stage I and II	41/41	0/41	12/24	≤5 cm	Stage I and II	36/36	0/36
Zhang Louqian	28/28	3.2 ± 1.6	Stage I and II	0/28	NA	70/70	3.6 ± 1.3	Stage I and II	0/70	NA
Yong Tang	NA	NA	NA	20/20	NA	25	NA	NA	25/25	NA

**Table 6:** Assessment of study quality

Study	Quality indicators from the Newcastle-Ottawa scale									Score
	Selection				Comparability		Exposure/Outcome			
	1	2	3	4	5a	5b	6	7	8	
Suda Takashi [13]	Yes	Yes	No	Yes	Yes	Yes	Yes	Yes	Yes	8
Hsu C-P [12]	Yes	Yes	No	Yes	Yes	No	Yes	Yes	Yes	7
Wang, H [15]	Yes	Yes	No	Yes	Yes	Yes	Yes	Yes	Yes	8
Yano Motoki [10]	Yes	Yes	No	Yes	Yes	Yes	Yes	Yes	Yes	8
Lu Qiang [9]	Yes	Yes	No	Yes	Yes	Yes	Yes	Yes	Yes	8
Zhang Louqian [8]	Yes	Yes	No	Yes	Yes	Yes	Yes	Yes	Yes	8
Yong Tang [14]	Yes	Yes	No	Yes	Yes	No	Yes	Yes	Yes	7

**Fig. 2:**
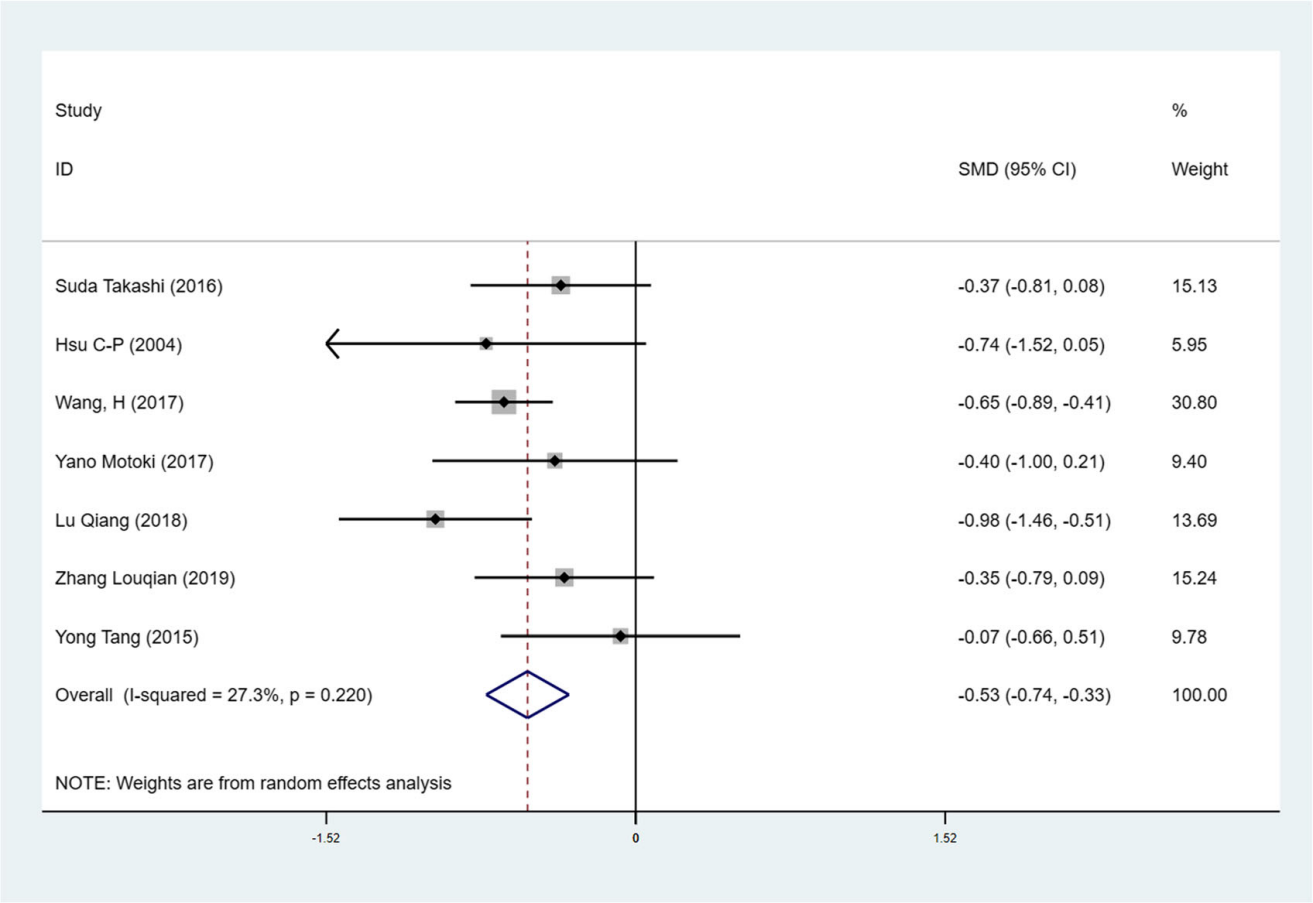
SVATS versus LVATS thymectomy, operative time (minutes)

**Fig. 3:**
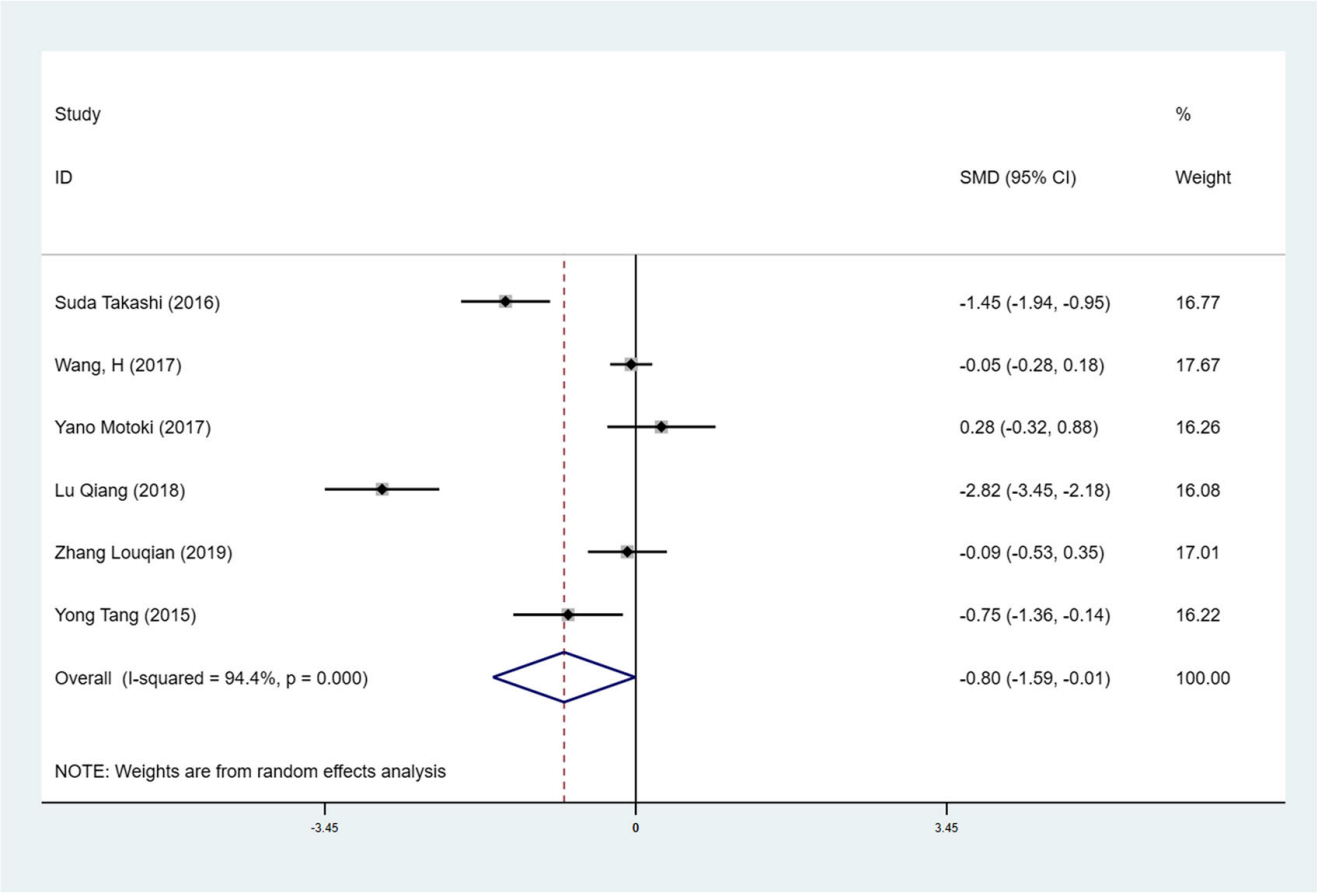
SVATS versus LVATS thymectomy, blood loss (ml)

**Fig. 4:**
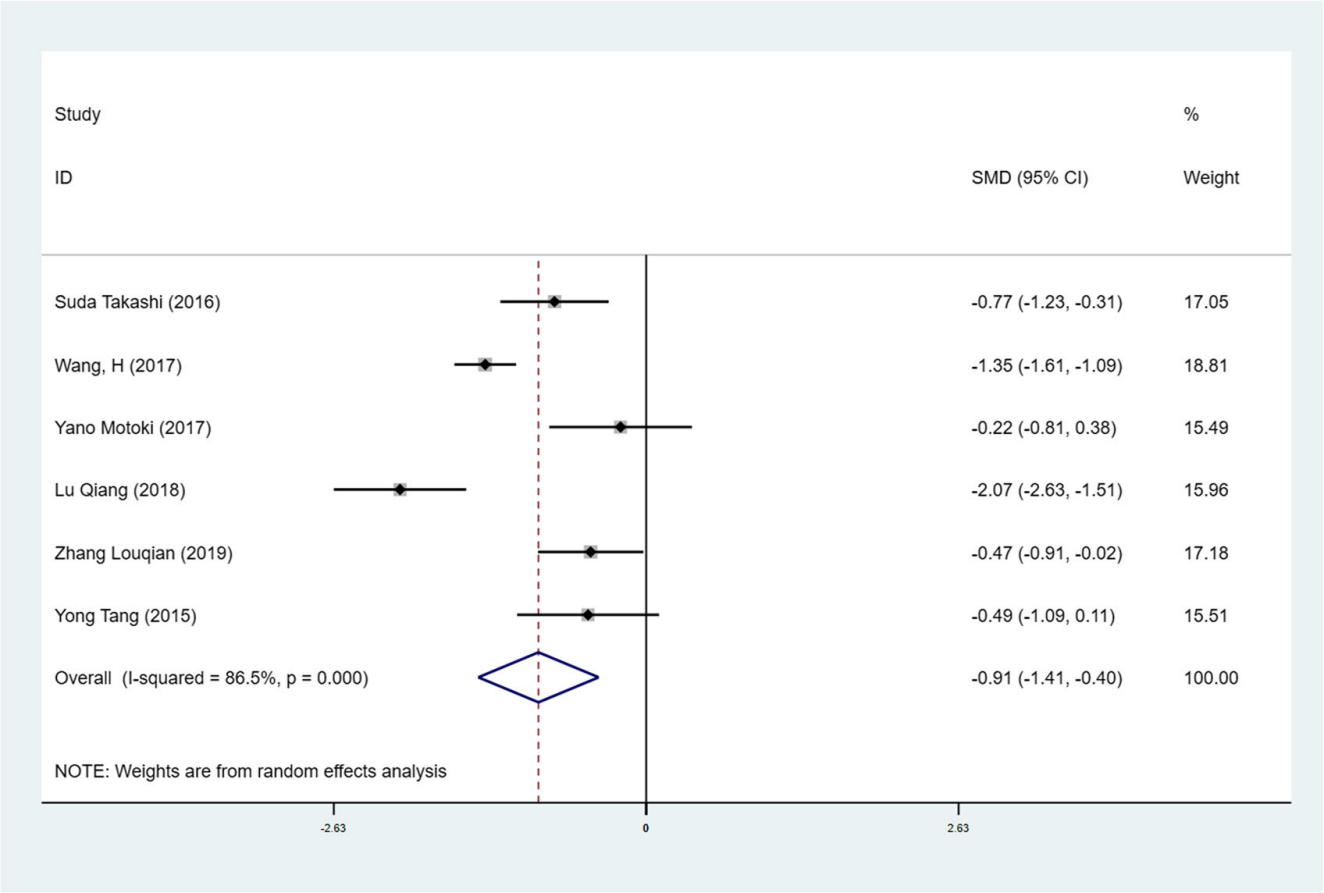
SVATS versus LVATS thymectomy, hospital stay (days)

**Fig. 5:**
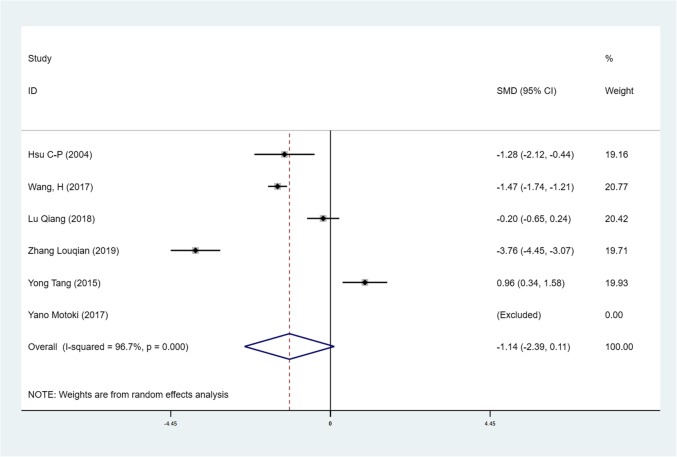
SVATS versus LVATS thymectomy, postoperative pleural drainage (days)

**Fig. 6:**
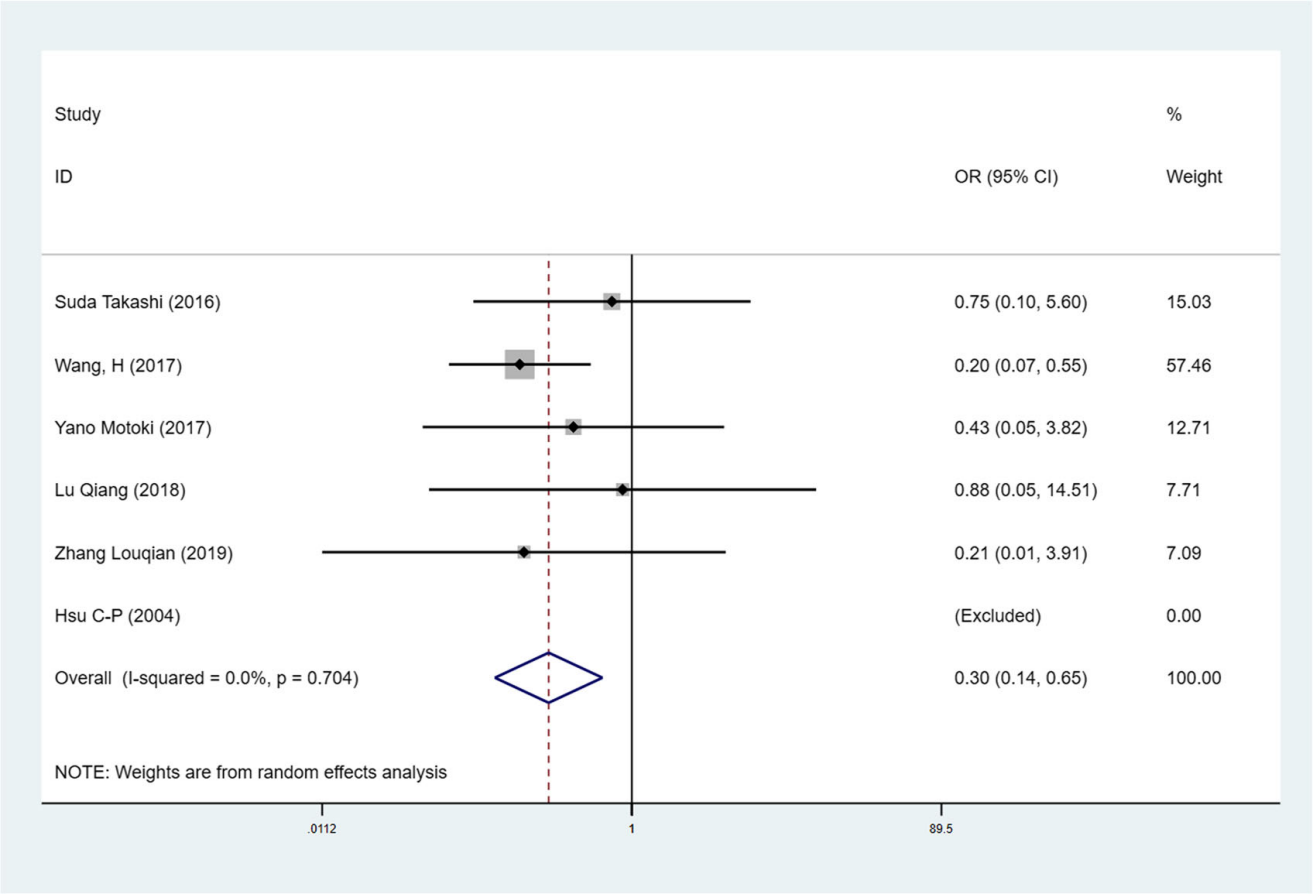
SVATS versus LVATS thymectomy, postoperative complications

**Fig. 7:**
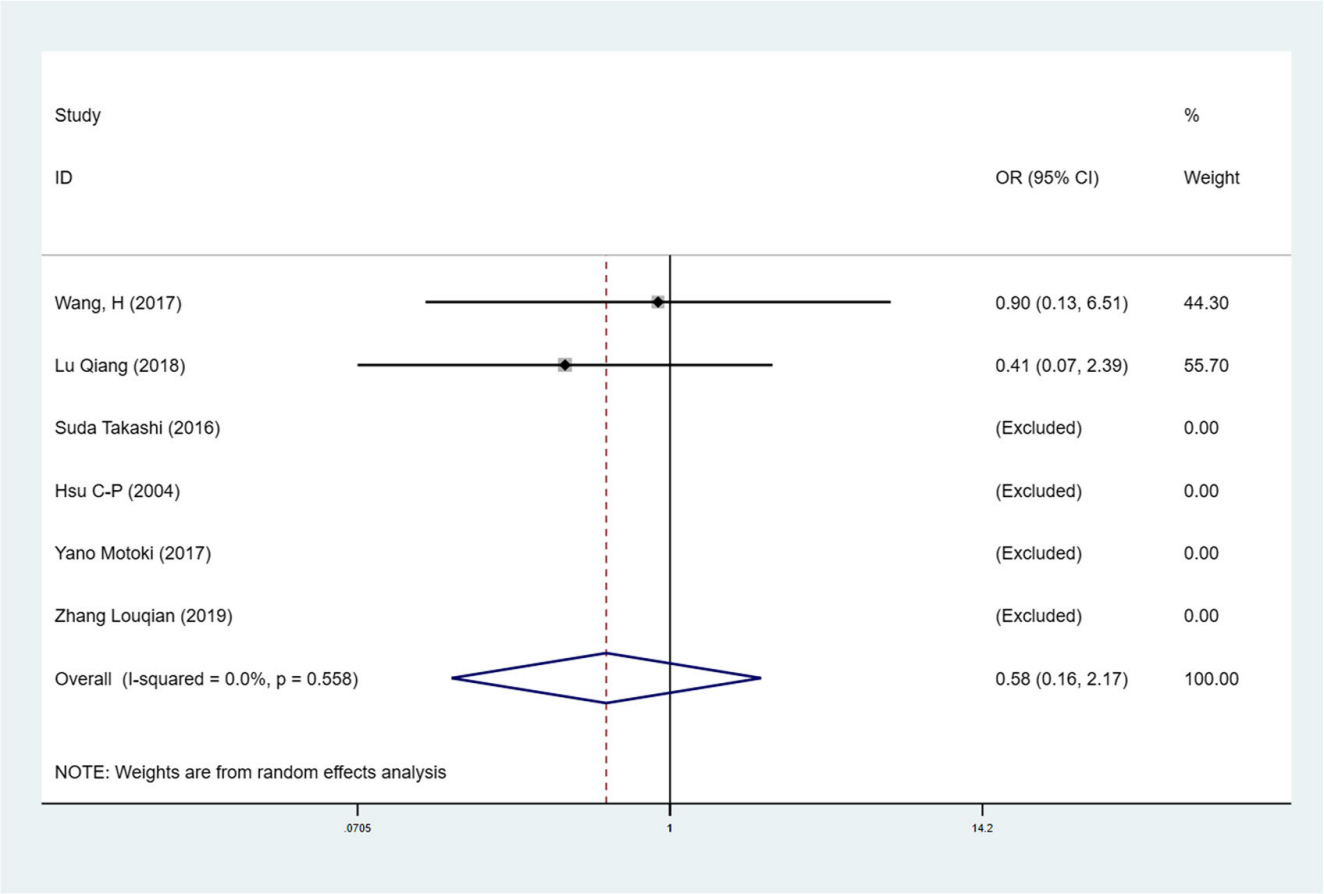
SVATS versus LVATS thymectomy, conversion to open

**Fig. 8:**
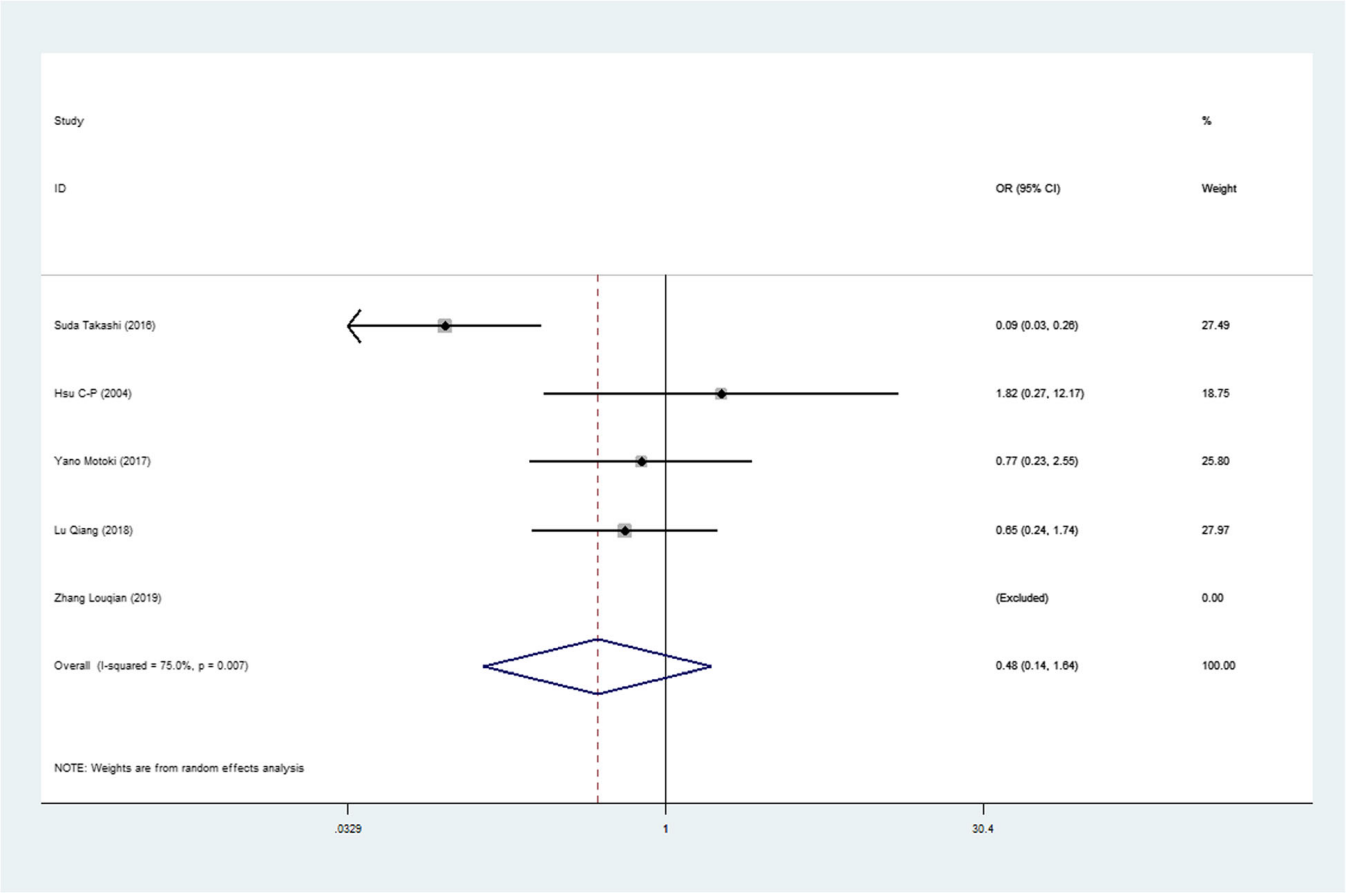
SVATS versus LVATS thymectomy, oncologic outcomes

We found mean operative time to be significantly less in patients in the SVATS group than in patients in the LVATS group (119.2 versus 138.8 min, standard difference = − 0.532, 95% confidence interval (CI): − 0.735 to − 0.329, p <0.001), as shown in Fig. 2. There was a significant difference between patients in the SVATS group and patients in the LVATS group about blood loss (46.8 versus 60.3 ml, standard difference = − 0.796, 95% CI: − 1.586 to − 0.007, *p* = 0.048), as shown in Fig. 3. Length of hospital stay was shorter for patients in the SVATS group (3.7 days for the SVSTS group versus 6.2 days for the LVATS group, standard difference = − 0.906, 95% CI: − 1.412 to − 0.400, *p* < 0.001), as shown in Fig. 4. There was no significant difference between postoperative pleural drainage stay (1.5 days for the SVATS group versus 2.6 days for the VATS group, standard difference = − 1.143, 95% CI: − 2.394 to 0.107, *p* = 0.073), as shown in Fig. 5. There was a significant difference in terms of postoperative complications (11 in the SVATS group versus 24 in the LVATS group, OR = 0.299, 95% CI: 0.137–0.653, *p* = 0.002), as shown in Fig. 6. There was no significant difference between conversion to open (4 for the SVATS group versus 6 for the VATS group, OR = 0.482, 95% CI, 0.156 to 2.166, *p* = 0.420), as shown in Fig. 7. There was no significant difference between oncologic outcomes (32 for the SVATS group versus 67 for the VATS group, OR = 0.582, 95% CI, 0.142 to 1.635, *p* = 0.241), as shown in Fig. 8.

## Discussion

In this study, we observed that the statistically significant clinical outcomes were decreased operative time, blood loss, postoperative complications, and shorter hospital stay, both of which favored the SVATS group. We found no differences in postoperative pleural drainage stay and conversion to open between the two groups. Most studies showed that SVATS have less pain after surgery than LVATS [8, 9, 13, 15]. Our results indicate that SVATS may be a better choice for thymectomy.

In LVATS thymectomy, surgeons may encounter a poor view of mediastinal fat and phrenic nerve at the contralateral side, and it is almost impossible to obtain adequate removal of the mediastinal fat on the contralateral side [16, 17]. Comparing to LVATS, SVATS can provide better surgical views in the upper pole of thymus and bilateral phrenic nerves [11, 18, 19], which might reduce the incidental injury, and this is essential for adequate bilateral mediastinal fatty tissue dissection. Although the bilateral approach provided adequate exposure of the anterior mediastinum, a higher number of incisions is needed, which may increase operative trauma [17]. Our findings on shorter hospital stay and less blood loss agree with those reported by Suda et al., who encourage surgeons to try this approach as one method of performing VATS thymectomy [13]. This study only included patients with thymoma, Masaoka phase I and II, so both methods can achieve R0 resection [9, 10, 12, 13].

However, all studies about subxiphoid VATS are small-scale and retrospective, further large-scale prospective study is necessary to confirm the benefit of SVATS. Our meta-analysis was limited by the inclusion of only nonrandomized, retrospective studies. Besides, there is a paucity of long-term follow-up data for patients who have undergone thymectomy for myasthenia gravis and thymomas. Our analysis was constrained by the inability to perform propensity matching because of small aggregate sample size and difficulty in obtaining individual patient information from the included studies. These factors led to increased heterogeneity within the analysis. In the end, we need to consider another factor that whether to have a total thymectomy or a subtotal thymectomy or a partial thymectomy. We mostly advocate total thymectomy in all cases of myasthenia gravis and thymomas.

## Conclusion

Based on the findings of this meta-analysis, we conclude that for selected patients, SVATS thymectomy is safe and may achieve better perioperative outcomes to those of LVATS thymectomy operations.

## Data Availability

The datasets generated and analyzed during the current study are available from the corresponding author on reasonable request.

## Abbreviations

VATS: video-assisted thoracoscopic surgery
SVATS: subxiphoid video-assisted thoracoscopic surgery
LVATS: lateral video-assisted thoracoscopic surgery
MG: myasthenia gravis
